# Association of Attitudes and Beliefs towards Antiretroviral Therapy with HIV-Seroprevalence in the General Population of Kisumu, Kenya

**DOI:** 10.1371/journal.pone.0004573

**Published:** 2009-03-04

**Authors:** Craig R. Cohen, Michele Montandon, Adam W. Carrico, Stephen Shiboski, Alan Bostrom, Alfredo Obure, Zachary Kwena, Robert C. Bailey, Rosemary Nguti, Elizabeth A. Bukusi

**Affiliations:** 1 Departments of Obstetrics, Gynecology and Reproductive Sciences, University of California San Francisco, San Francisco, California, United States of America; 2 Center for AIDS Prevention Studies, University of California San Francisco, San Francisco, California, United States of America; 3 Center for Epidemiology and Biostatistics, University of California San Francisco, San Francisco, California, United States of America; 4 Center for Microbiology Research, Kenya Medical Research Institute, Kisumu, Kenya; 5 Division of Epidemiology and Biostatistics, University of Illinois at Chicago, Chicago, Illinois, United States of America; 6 Department of Statistics, University of Nairobi, Nairobi, Kenya; National AIDS Research Institute, India

## Abstract

**Background:**

Since antiretroviral therapy (ART) became available in the developed world, the prevalence of unprotected sex and the incidence of sexually transmitted infections (STIs) and HIV have increased. We hypothesized that a similar phenomenon may be occurring in sub-Saharan Africa concomitant with the scale-up of HIV treatment.

**Methods:**

We conducted a general population-based survey in Kisumu, Kenya. Participants completed an interview that included demographics as well as ART-related attitudes and beliefs (AB) and then underwent HIV serological testing. Exploratory and confirmatory factor analyses of AB about ART indicated two factors: 1) ART-related risk compensation (increased sexual risk taking now that ART is available); and 2) a perception that HIV is more controllable now that ART is available. Logistic regression was used to determine associations of these factors with HIV-seroprevalence after controlling for age.

**Findings:**

1,655 (90%) of 1,844 people aged 15–49 contacted, including 749 men and 906 women, consented to participate in the study. Most participants (n = 1164; 71%) had heard of ART. Of those who had heard of ART, 23% believed ART was a cure for HIV. ART-related risk compensation (Adjusted (A)OR = 1.45, 95% CI 1.16–1.81), and a belief that ART cures HIV (AOR = 2.14, 95% CI 1.22–3.76) were associated with an increased HIV seroprevalence in men but not women after controlling for age. In particular, ART-related risk compensation was associated with an increased HIV-seroprevalence in young (aged 15–24 years) men (OR = 1.56; 95% CI 1.12–2.19).

**Conclusions:**

ART-related risk compensation and a belief that ART cures HIV were associated with an increased HIV seroprevalence among men but not women. HIV prevention programs in sub-Saharan Africa that target the general population should include educational messages about ART and address the changing beliefs about HIV in the era of greater ART availability.

## Introduction

In 2007, almost two-thirds of all HIV-positive persons were living in sub-Saharan Africa, and an estimated 1.7 million adults and children became infected that same year, more than in all the other regions in the world combined [1]. Kenya has an advanced HIV epidemic with an estimated 1.2 million persons, roughly 6.1% of the total population, living with HIV [1]. Since the Declaration of Commitment issued by the United Nations General Assembly Special Session on AIDS in 2001, access to anti-retroviral therapy (ART) and HIV/AIDS care in developing countries has become a global priority. The Global HIV Prevention Working Group, the World Health Organization strategy, “Treating 3 million by 2005,” and other HIV treatment initiatives have recommended the integration of HIV prevention and treatment services. However, there is little research available to guide the design and delivery of evidence-based prevention services in the era of ART [2], [3].

In sub-Saharan Africa, ART coverage among those eligible increased from 2% in 2003 to 17% at the end of 2005, and access to ART continues to increase rapidly [4]. ART in the public sector in Kenya was first piloted in late 2003 at five sites. Of the estimated 250,000 persons requiring ART in Kenya, the number of persons receiving ART increased from 65,000 (26%) to 110,000 (44%) between the start of 2006 and 2007 (Dr. Lyndon Marani, ART program officer, National AIDS and STD Control Program, Kenya, personal communication). In Kisumu, the location of this investigation, of the estimated 37,784 persons eligible for ART 4,972 (13%) and 10,464 (28%), were receiving ART by the start of 2006 and 2007, respectively (Dr. Steven Onditi, Kisumu District Medical Officer of Health Kisumu, personal communication).

An epidemiologic model of HIV/AIDS, calibrated to sub-Saharan Africa, predicted that treatment-centered programs would produce smaller benefits than prevention-centered programs, but that integrating programs of prevention and treatment would have a synergistic effect and yield optimal reductions in HIV incidence and mortality [5]. Enhanced availability of HIV treatment programs will likely facilitate HIV prevention by reducing stigma, increasing HIV testing rates, and creating a common infrastructure for HIV services [6]. At the same time, an overemphasis on treatment programs could also detract from prevention efforts and lead to reduced public concern about HIV and increased HIV risk behaviors [7], [8].

Numerous studies in the United States and Europe have identified an upward trend in risky sexual behaviors since the introduction of ART in 1996 [9]. More specifically, an association has been identified between decreased concern about HIV due to ART availability and unprotected sex [10], [11], [12], [13], [14]. Lending further support to these findings, a longitudinal study of men who have sex with men in the Netherlands observed that participants who perceived HIV/AIDS as less threatening since ART availability had a higher incidence of sexually transmitted infections (STIs) and HIV [15].

Investigations of ART-related attitudes and beliefs (AB) in sub-Saharan Africa are limited. A descriptive study of beliefs about HIV/AIDS and ART among 105 HIV clinic patients in Soweto, South Africa found that participants' impressions of ART were overly optimistic, for example that ART could cure HIV (49%) and that ART would not cause side effects (36%) [16]. Although several studies have found that regular access to treatment facilities may promote safer sex for those on ART [17], [18], no study to date in Africa has examined AB about ART in the general population and the extent to which ART-related AB are associated with HIV-seroprevalence. Therefore, we conducted a population-based survey to determine if AB about ART are associated with HIV-seroprevalence during the scale-up of HIV treatment in sub-Saharan Africa. We specifically hypothesized that AB about ART indicating decreased concern about HIV and increased sexual risk-taking due to ART availability would be associated with an increased risk of testing HIV-seropositive. In addition, we reasoned that if the association between ART-related AB and HIV-seroprevalence reflected an increased likelihood of sexual risk taking, then this association would be strongest among adolescents and young adults – a group more likely to have become sexually active since the roll out of ART.

## Methods

### Study site

The study took place in the Municipality of Kisumu, situated in western Kenya on Lake Victoria. Kisumu is Kenya's third largest city with a population of approximately 400,000. The majority of Kisumu's residents belong to the Luo ethnic group, one of the largest tribes in Kenya.

### Study design

The first phase of the study consisted of rapid community assessment with social mobilization of community leaders to gather their input and support for the study. Focus group discussions with community members were conducted to assess local understanding of ART for development of our study instrument. The acceptability of blood, urine and vaginal swabs for specimen collection was also assessed, and standardized methods of collection were developed. Finally, data collection instruments were modified and pre-tested. The questionnaires were written in English and then translated into the other commonly spoken languages in Kisumu: Kiswahili and Dholuo. The final study protocol, consent and questionnaire were approved by the ethical committees of the Kenya Medical Research Institute and the University of California, San Francisco. Data collection took place from July–October 2006. Sampling methods from the Four African Cities Study conducted in Kisumu in 1997 were adopted and replicated as much as possible so that sexual behavioral and STI/HIV data from the two studies would be comparable [19].

### Survey of the general population

Households were selected by two-stage sampling, and all persons aged 15–49 years who slept in the house the night before the first visit by the research team were eligible for the study. Within Kisumu, 23 sentinel clusters were used that were determined to be representative of the population in this municipality. Sentinel clusters were supplemented with the systematic selection of an additional 17 census enumeration areas within the municipality of Kisumu, resulting in a total of 40 clusters for study. The Municipality of Kisumu includes both the city center as well as surrounding rural areas. Of our 40 clusters, 22 were considered urban and 18 rural. All households in these clusters were enumerated, and the total number of households in our sampling frame was determined by the target sample size (2000) adjusted for a 20% non-response rate divided by the average number of eligible participants (2.15) per household. Households were selected by systematic random sampling: a random ‘start point’ was chosen between 1 and 4, with every fourth household sampled thereafter.

Each household was visited a day or two prior to the study by the community mobilizer, who informed selected households about the study team's upcoming visit. The household was then visited by the study team. If eligible participants were not at home, the study team made at least 2 additional visits to the household. If no one was found at home on all three visits, this household was replaced with the next household number. As shown in Figure S1, of the 1,210 households in the original sampling frame, 708 were contacted (59%) and 645 (53%) had at least one household member who participated in the study. Of the 502 households that needed replacement, 219 households (43%) participated in a second round of sampling. This resulted in a total of 864 participating households. Of the 3,376 eligible persons in these households, 1,844 (55%) were contacted and asked to enroll and 1532 (45%) were not at home during the study visit. Of the 1,844 who were contacted, 1655 (91%) people (749 men and 906 women aged 15–49) enrolled in the study and 189 (9%) refused to enroll.

After giving written informed consent, each participant was interviewed on sociodemographic characteristics, HIV knowledge, ART-related AB, sexual history with spousal and non-spousal partners, history of STIs, HIV testing history, and HIV risk perception. The interviewer recorded participant responses in a pre-programmed questionnaire in the personalized digital assistant (PDA). Study interviews and specimen collections were carried out at a private location in or nearby the homes of the study participants. After the interview, study team members requested that participants provide a peripheral venous blood sample. In total, 1508 participants (91%) provided a blood sample (Figure S1), which was tested for HIV. Study participants with symptoms and/or signs suggestive of a STI were treated immediately by the study nurse using the Kenyan guidelines for STI syndromic management. HIV testing results were linked to interview data using a separate “link” identification number to protect confidentiality. Beginning approximately one to two weeks after specimen collection, trained testing counselors offered HIV counseling and test results both within the selected clusters and at a central location in town. Those who tested HIV-positive were referred to HIV care and treatment facilities.

### Survey measures

Data were collected on background variables including: age, gender, education, employment, religion, marital status, place of birth, socioeconomic status, recent travel, and history of drug or alcohol use. The section on HIV and ART-related AB was adapted from prior studies and modified to fit the local context [10], [15], [20]. The 13-item section regarding ART-related AB assessed whether the participant was less concerned about HIV/AIDS or reported that there was less need for safer sex since the availability of ART. Possible responses included: “agree”, “unsure”, or “disagree.” If a participant did not have an answer to a particular question the response was left blank. The questionnaire underwent pre-testing with community members, who were encouraged to give their perceptions regarding the meaning of each question and voice concerns about any areas of confusion. Informed by this process, the questionnaire was refined to ensure that it was culturally appropriate, easy to understand, and properly adapted from English into the two local languages.

### Laboratory procedures

Laboratory HIV testing of venous blood was performed according to Kenyan National guidelines for voluntary counseling and testing. Specimens were first tested using parallel rapid assays (Rapid Uni-Gold™, Trinity Biotech, Ireland; and Determine™, Inverness Medical Innovations, Delaware, USA). The presence of two positive rapid assays was defined as HIV seropositive, and two negative rapid assays was defined as HIV seronegative. Discordant or indeterminate rapid assay results were resolved using HIV ELISA (Vironostika HIV Uni-Form II Ag/Ab).

### Data analysis

We began with an exploratory factor analysis (EFA) in order to determine the structure of the 13 items that assessed ART-related attitudes and beliefs. EFA allows for the estimation of the shared variance in items that is explained by a given factor while partialing out the influence of measurement error. The ability to examine only the shared variance in items is the primary advantage of EFA when compared to principal components analysis, which examines all the variance in items (including measurement error) [21]. Because the 13 items allowed for three response options (agree = 2, unsure = 1, or disagree = 0) the EFA was conducted using MPlus version 5.1 (Muthén & Muthén, Los Angeles, CA), which allows one to conduct an EFA with categorical indicators using the weighted least squares-mean and variance-adjusted estimator [22]. Furthermore, since knowledge of ART was a prerequisite to respond to these questions, we only used data from the 71% of participants that responded that they had heard of ART in the EFA. We interpreted the results of this EFA by examining the number of eignvalues that were greater than 1.50 as well as the degree to which items loaded strongly (0.40 or higher) on a single factor with promax rotation. Next, we conducted a confirmatory factor analysis (CFA) to examine if specifying the factor structure from the EFA (where items only loaded on one factor) was an adequate fit for the data. For the present investigation, we utilized multiple indices of model fit: a chi-square to degrees of freedom ratio (χ^2^/df) less than three, comparative fit index (CFI) values greater than 0.95, root mean square error of approximation (RMSEA) values less than 0.06, and weighted root-mean-square residual (WRMR) values less than 0.90 [23], [24]. Informed by the results of the EFA and CFA, we created composite scores for the factors. The associations of these composite scores with HIV-seroprevalence were examined using logistic regression in SPSS 15.0 (SPSS Inc., Chicago, Illinois, USA). Given the cultural context of sub-Saharan Africa where men are generally afforded greater autonomy and independence than women, we theorized that the associations of ART-related attitudes and beliefs with HIV-seroprevalence may vary as a function of gender [25]. Consequently, separate logistic regression analyses were conducted for men and women with age as a covariate. Individuals who had prior knowledge of their HIV serostatus were not included in these analyses. In order to facilitate the interpretation of the adjusted odds ratio (AOR), the composite scores for factors of ART-related attitudes and beliefs were transformed into z-scores (*M* = 0, *SD* = ±1). Where significant associations between ART-related attitudes and beliefs with HIV-seroprevalence were observed within a given gender, we conducted follow-up logistic regression analyses to examine whether this effect varied by age category (i.e., 15–24, 25–29, 30–39 and 40–49 years).

Sociodemographic characteristics were compared by HIV-serostatus after stratification by gender, and controlling for age by category (as above) using logistic regression; odds ratios (OR) and 95% confidence intervals (CI) were calculated.

## Results

### Association of sociodemographic factors with HIV seroprevalence

In general, sociodemographic findings were similar between the 1,508 participants who gave blood for HIV serological testing and the 147 who refused testing (data not shown). Of those who underwent HIV serological testing, 25% of participants were between the ages of 15–19 years, 31% between 20–24 years, 17% between 25–29 years, 18% between 30–39 years and 9% between 40–49 years. Most participants were Luo (77%) and Christian (95%). The majority (53%) had only a primary school education, 36% had a secondary school education, 8% had college or university education, and 3% had no formal education. Twenty five percent of women and 16% of men who provided blood samples were HIV seropositive (OR = 1.7, 95% CI 1.3–2.8). HIV seroprevalence increased significantly with age (Table 1). Among those who tested HIV-seropositive, 9 of 109 (11%) men and 28 of 204 (14%) women reported prior knowledge of their positive HIV serostatus. For men, being single was associated with a decreased HIV seroprevalence, while for women being separated/divorced or widowed was significantly associated with an increased HIV seroprevalence in comparison to those who were married. Women, but not men, with primary and secondary school education had a higher HIV seroprevalence than those with greater education. Employment had a protective effect for women while for men employment was associated with a non-significant increased HIV seroprevalence. Religious affiliation for men and women and identified ethnicity for men were not associated with HIV. However, Luo women were two-times more likely to be HIV infected than women of other ethnic groups. Alcohol use for men and women and other drug use for men correlated with increased HIV infection. Although we did not directly ask about income, presence of electricity at one's home was associated with a lower HIV seroprevalence. As expected, a history of a positive HIV test strongly correlated with HIV test results performed as part of the study. Eleven percent of men and 11% of women who reported testing HIV negative during their last HIV test seemingly seroconverted. Women who reported that they did not return for prior HIV test results had a higher HIV seroprevalence than those who had never been tested (Table 1).

**Table 1:** The association of sociodemographic factors and HIV−seroprevalence stratified by sex in Kisumu.

Variable	Men			Women		
	HIV−/ve	HIV+/ve	OR, 95% CI*	HIV−/ve	HIV+/ve	OR, 95% CI*
Age category (years)						
15–19	172 (96%)	7 (4%)	1.0	173 (90%)	19 (10%)	1.0
20–24	158 (87%)	24 (13%)	3.8 (1.8–8.3)	217 (80%)	54 (20%)	2.2 (1.2–4.0)
25–29	101 (78%)	29 (22%)	7.3 (3.4–15.5)	95 (70%)	40 (30%)	3.8 (1.9–7.6)
30–39	94 (80%)	24 (20%)	6.5 (2.7–15.6)	91 (59%)	63 (41%)	6.3 (3.4–11.6)
40–49	46 (65%)	25 (35%)	13.5 (5.5–33.4)	48 (63%)	28 (37%)	5.3 (2.7–10.5)
What is your marital status?						
Married	146 (71%)	61 (30%)	1.0	275 (78%)	79 (22%)	1.0
Single	231 (93%)	18 (7%)	0.3 (0.1–0.6)	127 (86%)	21 (14%)	1.0 (0.5–2.0)
Separated/divorced	16 (76%)	5 (24%)	0.7 (0.2–2.1)	23 (61%)	15 (39%)	2.3 (1.2–4.3)
Widowed	2 (67%)	1 (33%)	0.8 (0.1–7.7)	14 (34%)	27 (66%)	4.4 (2.2–9.1)
Currently married?						
No	249 (91%)	24 (9%)	1.0	164 (72%)	63 (28%)	1.0
Yes	146 (71%)	61 (29%)	2.6 (1.3–5.2)	275 (78%)	79 (22%)	0.5 (0.3–0.8)
Educational level						
Primary	142 (77%)	43 (23%)	2.1 (0.8–5.3)	220 (72%)	85 (28%)	5.6 (1.8–17.6)
Secondary	189 (85%)	33 (15%)	1.6 (0.6–4.1)	166 (78%)	46 (22%)	3.7 (1.1–12.4)
College/University	63 (90%)	7 (10%)	1.0	43 (96%)	2 (4%)	1.0
Currently employed?						
No	198 (89%)	25 (11%)	1.0	272 (76%)	85 (24%)	1.0
Yes	197 (77%)	60 (23%)	1.5 (0.9–2.6)	167 (75%)	57 (25%)	0.7 (0.5–0.97)
Religion						
Catholic	122 (84%)	24 (16%)	N/A	104 (72%)	41 (28%)	N/A
Other Christian	248 (81%)	58 (19%)		319 (77%)	94 (23%)	
Muslim	20 (87%)	3 (13%)		15 (88%)	2 (12%)	
No religion	2 (100%)	0 (0%)		0 (0%)	3 (100%)	
Other	3 (100%)	0 (0%)		1 (33%)	2 (67%)	
Christian						
No	25 (89%)	3 (11%)	1.0	16 (70%)	7 (30%)	1.0
Yes	370 (82%)	82 (18%)	1.6 (0.3–8.0)	423 (76%)	135 (24%)	0.8 (0.3–2.2)
Luo						
No	81 (84%)	15 (16%)	1.0	108 (82%)	23 (18%)	1.0
Yes	314 (82%)	70 (18%)	1.4 (0.7–2.8)	331 (74%)	119 (26%)	2.0 (1.2–3.2)
Location						
Rural	189 (80%)	48 (20%)	1.6 (0.9–2.8)	207 (74%)	72 (26%)	1.1 (0.7–1.6)
Urban	206 (85%)	37 (15%)	1.0	232 (77%)	70 (23%)	1.0
Had alcohol last four weeks?						
No	235 (88%)	32 (12%)	1.0	395 (78%)	115 (22%)	1.0
Yes	160 (75%)	53 (25%)	1.9 (1.2–3.0)	44 (62%)	27 (38%)	2.1 (1.3–3.5)
Drug use ever?						
No	236 (88%)	31 (12%)	1.0	397 (77%)	122 (23%)	1.0
Yes	159 (75%)	54 (25%)	2.2 (1.3–3.7)	42 (68%)	20 (32%)	1.5 (0.8–2.8)
Do you have electricity?						
No	261 (80%)	66 (20%)	1.0	284 (72%)	113 (28%)	1.0
Yes	134 (88%)	19 (12%)	0.6 (0.3–0.7)	155 (84%)	29 (16%)	0.5 (0.3–0.7)
Of those who had prior HIV testing, HIV result at last testing?						
Positive	3 (30%)	9 (70%)	13.5 (4.2–42.5)	1 (3%)	28 (97%)	176 (21–1504)
Negative	194 (89%)	23 (11%)	1.0	288 (87%)	44 (13%)	1.0
Did not get results slip	4 (67%)	2 (33%)	5.0 (0.5–52.5)	18 (75%)	6 (25%)	3.2 (1.1–9.8)
Length of time in Kisumu?						
<1 year	40 (83%)	8 (17%)	1.0	58 (88%)	8 (12%)	1.0
1 to 5 years	102 (87%)	15 (13%)	0.6 (0.2–1.8)	125 (82%)	28 (18%)	1.5 (0.6–3.4)
5+ years	253 (80%)	62 (20%)	0.9 (0.4–2.0)	256 (71%)	106 (29%)	1.9 (0.9–4.2)

* Adjusted for age category.

### ART-related attitudes and beliefs

Of the 1,508 participants who were tested for HIV, most (71%) had heard of ART, with no significant differences by sex (men: 71% vs. women: 71%, p = .53). Of those who had heard of ART, 87% thought that ART would be available to them if needed and 77% knew of a health facility where they could get ART. Nearly all (98%) agreed that ART prolongs life, while 23% believed that ART was a cure for HIV/AIDS and 26% thought that ART had no side effects. Using the 13 items that assessed ART-related attitudes and beliefs, we conducted an EFA and observed two eignvalues that were greater than 1.50 (eignvalues = 2.50 and 4.67). This suggests that two factors most adequately summarize these data. Of the 13 items, 11 loaded strongly (≥0.40) on a single factor in the EFA. Two items (“ART cures HIV/AIDS” and “ART increases one's sexual drive”) did not load strongly (<0.40) on either factor. As a result, these items were not included in the CFA. For the CFA, we observed that the model was an adequate fit for the data (χ^2^/df = 2.92, CFI = .96, RMSEA = 0.04, WRMR = 1.09). All four items for the first factor and all seven items for the second factor loaded strongly (≥0.40). As summarized in Table 2, the 4-item factor appeared to reflect perceptions that HIV/AIDS is more controllable since the availability of ART (Cronbach's α = .61), while the 7-item factor seemed to measure ART-related risk compensation (Cronbach's α = .66). Because it was not easily summarized by either of the factors, we included the belief that “ART cures HIV/AIDS” as a single item predictor in subsequent analyses. We reasoned that this fixed, incorrect belief may have important implications for sexual risk taking behavior and HIV-seroprevalence.

**Table 2:** Factors derived from ART-related attitudes and beliefs.

**Factor 1: Belief that HIV is a more controllable disease due to ART availability (α = .61)**
Now that ART is available, HIV is less serious than it used to be.
Now that ART is available, it is more important for people to know their HIV status.
Now that ART is available, HIV/AIDS is a controllable disease.
Now that ART is available, people are more willing to get tested for HIV.
**Factor 2: ART-related risk compensation (α = .66)**
Now that ART is available, people do not need to be as concerned about becoming HIV-positive.
Now that ART is available, condom use during sex is less necessary.
Now that ART is available, you are less worried about HIV infection.
Now that ART is available, you are more likely to have more than one sexual partner.
Now that ART is available, you are more willing to take a chance of getting infected or infecting someone else with HIV.
Now that ART is available, someone who is HIV-positive does not need to worry as much about condom use.
Now that ART is available, you are more likely to have sex without a condom.

### Association of ART-related attitudes and beliefs with HIV-seroprevalence

After controlling for age, ART-related risk compensation (Table 2) was associated with increased HIV-seroprevalence for men (AOR = 1.45, 95% CI 1.16–1.81) but not for women (AOR = 1.08, 95% CI .89–1.31). The perception that HIV/AIDS is more controllable since the availability of ART (Table 2) was not statistically associated with HIV-seroprevalence for men (AOR = 1.28, 95% CI .97–1.69) or women (AOR = .99, 95% CI .82–1.20) after controlling for age. These AORs reflect the increase in the odds for testing HIV-positive that is associated with one standard deviation increase in the specified factor. Finally, a belief that ART cures HIV/AIDS was associated with increased HIV-seroprevalence among men (AOR = 2.14, 95% CI 1.22–3.76) but not women (AOR = 1.43, 95% CI .93–2.21) after controlling for age.

Because we hypothesized that the effect of any association would be greatest for adolescents and young adults we performed further analysis stratified by age categories for men. ART-related risk compensation was associated with increased HIV-seroprevalence among 15–24 year old men. In addition, a belief that ART cures HIV/AIDS was associated with a markedly elevated HIV-seroprevalence among 25–29 year old men (Table 3). Results of the age-controlled and age-stratified analyses were virtually unchanged when the 7 men and 27 women who reported a previous HIV-positive test and were confirmed HIV-positive in the study were excluded (data not shown).

**Table 3:** Odds ratios for the association between ART-related attitudes and beliefs, and HIV seroprevalence among men of different age categories.

	15–24 Years Old (n = 233)	25–29 Years Old (n = 99)	30–39 Years Old (n = 90)	40–49 Year Old (n = 58)
	OR (95% CI)	OR (95% CI)	OR (95% CI)	OR (95% CI)
HIV is more controllable since ART factor*	1.30 (0.79–2.17)	1.45 (0.87–2.41)	1.07 (0.59–1.94)	1.30 (0.69–2.43)
ART-related risk compensation factor*	1.56 (1.12–2.19)	1.35 (0.80–2.28)	1.45 (0.87–2.40)	1.32 (0.83–2.11)
“ART Cures HIV/AIDS”	1.58 (0.54–4.60)	3.43 (1.17–10.08)	2.27 (0.72–7.16)	1.56 (0.43–5.58)

* See Table 2 for attitudes and beliefs included in each factor.

### Changes in HIV-seroprevalence between 1997 and 2006

Because we replicated the sampling methodology used in the Four African Cities Study performed in 1997, we were able to assess changes in HIV-seroprevalence in the Kisumu population between the two time periods. Nevertheless, the percentages of eligible individuals who participated and gave a blood specimen for HIV serological testing in sampled households were significantly lower in 2006 than in 1997 for women (47% vs. 75%) and for men (42% vs. 61%) (Table 4). Between 1997 and 2006, the prevalence of HIV declined from 30% to 25% in women and 20% to 17% in men [26]. HIV seroprevalence had the greatest decline in women 15–29 years of age with a less pronounced decline in men 25–39 years of age (Figure S2a & S2b) [19], [26]. The HIV seroprevalence was reduced by 57% in 15–19 year old women, 47% in 20–24 year old women and 19% in 25–29 year old women. Among women aged 30–39 and 40–49 years, HIV seroprevalence increased by 37% and 95%, respectively. Among men, HIV seroprevalence was unchanged for 15–19 year olds, with a 12% increase in 20–24 year olds and a 25% increase in 40–49 year olds. For men 25–29 and 30–39 year old, HIV seroprevalence reduced by 24% and 29%, respectively.

**Table 4:** Comparison of individual response rates between 2006 and 1997 [19] by age categories for men and women in Kisumu, Kenya.

	Eligible Participants		Interview (% Eligible)		Blood Taken (% Eligible)	
	2006	1997	2006	1997	2006	1997
**Men**						
15–19 years	401	239	197 (49%)	194 (81%)	179 (45%)	146 (61%)
20–29 years	729	376	350 (48%)	319 (85%)	312 (43%)	242 (64%)
30–39 years	339	259	128 (38%)	211 (81%)	118 (35%)	154 (59%)
40–49 years	158	140	74 (47%)	105 (75%)	71 (45%)	84 (60%)
**All**	1627	1014	749 (46%)	829 (82%)	680 (42%)	626 (62%)
**Women**						
15–19 years	447	323	213 (48%)	281 (87%)	192 (43%)	226 (70%)
20–29 years	824	469	445 (54%)	427 (91%)	406 (49%)	368 (78%)
30–39 years	315	266	169 (54%)	234 (88%)	154 (49%)	196 (74%)
40–49 years	163	133	79 (49%)	118 (89%)	76 (47%)	103 (77%)
**All**	1749	1191	906 (52%)	1060 (89%)	828 (47%)	893 (75%)

## Discussion

To our knowledge, this is the first population-based study in the developing world to examine the AB about ART and their relationship with HIV seroprevalence. Previous studies in sub-Saharan Africa have focused on the ART-related AB of HIV-positive persons receiving HIV care. The data from this cross-sectional study cannot provide evidence for a causal relationship, but we are able to gain a general understanding of attitudes and beliefs about ART in Kisumu, Kenya, where access to ART is rapidly expanding, and the ways that these attitudes and beliefs may increase risk for HIV infection.

In general, the majority of participants had heard of ART and knew some basic information about it, but a sizeable minority had misinformation (that ART is a cure). The majority of those who had heard of ART thought that HIV was a less serious threat now that ART is available. However, the most widely agreed upon statement was that “knowing one's HIV status is more important now that ART is available.” This indicates that ART availability could provide a strong impetus for HIV testing and potentially encourage HIV prevention behaviors through awareness of HIV status [6].

Young people represent not only one of the highest risk groups for HIV acquisition and transmission, but also are the group most likely to have become HIV-infected during the period in which ART became available in Kisumu. In our study, young men who reported ART-related AB consistent with risk compensation and those who believed that ART was a cure for HIV/AIDS had a significantly higher adjusted odds of being HIV positive than other young men without these attitudes and beliefs. Why these findings were limited to the male study population cannot fully be explained by these data. However, several hypotheses may account for the different results found between men and women. In Kenyan culture, as in many cultures worldwide, men are more able to act on their attitudes and beliefs than women especially in regards to sexual activity [25]. Thus, for women the risk of acquiring HIV may be more a marker of their sexual partner's attitudes and beliefs than of their own [27], [28] and the indirect effect of economic deprivation [29]. Additionally, the association between ART-related beliefs and HIV may not represent a causal association between beliefs and risk of HIV acquisition, but rather a “risk-taking” personality that is characterized by indifference toward risk and risky behaviors. In fact, we presume many of the HIV infections discovered occurred prior to the roll-out of ART services in Kisumu that started approximately three years before onset of this study and before knowledge about ART was widespread in the general population. It is also possible that there are different perceptions of risk between the sexes [30], [31], and that a risk-taking subgroup of men were more likely to have HIV and also more likely to be reassured that ART makes HIV less threatening. Any of these reasons for the association between ART AB and HIV prevalence could ultimately fuel the HIV epidemic; men who knowingly or unknowingly are HIV-infected engaging in higher risk sexual activity will ultimately lead to greater HIV transmission.

Despite the fact that every effort was made to adopt sampling procedures from the Four African Cities Study our response rates for the present investigation were significantly lower [19, Table 4]; thus, comparisons must be made with some degree of caution. Nevertheless, the declines in HIV seroprevalence, especially among women, are heartening and may represent the effects of greater awareness about HIV [32], reduction in STIs [33] and change in sexual behavior [34]. The increased HIV seroprevalence in older women and men may represent a cohort effect (i.e., the higher prevalence in younger women in the 1997 survey are now reflected in the cohort almost 10 years older), or could alternatively demonstrate a worrying trend towards increased HIV seroconversion in this age group. It is also noteworthy that the reduction in HIV seroprevalence was less marked in men. This may be the result of continued high risk exposure among men in comparison to women, and/or the fact that the majority of prevention efforts in Kisumu have focused on young women. Alternatively, men were less likely to be at home and were more challenging to recruit for this study; thus, men who enrolled may represent a higher risk group than those we could not locate for this study perhaps due to an illness, such as HIV that caused men to stay at home. With male circumcision services becoming more widely available in Kisumu, it is likely that the HIV seroprevalence will decline further in men over the next decade [35], [36], [37]. Nevertheless, it is important to interpret the comparison of these two HIV-seroprevalence estimates from 1997 and 2006 with some caution. Although differences between the1997 to 2006 Kisumu data may reflect true changes in HIV-seroprevalence, it is also plausible that these differences are attributable to variability in sampling procedures for these studies. We also suspect that growth and urbanization of Kisumu has led to a decreased likelihood of contacting eligible participants at home. Over and above these limitations, improved care of people living with HIV and increased availability of ART in Kisumu are likely important factors contributing to recently observed declines in HIV-related mortality [38]. Similarly, we can hope for a further decline in new HIV infections as access to ART continues to increase [39], [40]. Findings from the present investigation underscore the need for enhanced efforts to prevent HIV seroconversion in sub-Saharan Africa, especially among young men and their sexual partners who may be more vulnerable to contracting HIV due to attitudes that reflect ART-related risk compensation or incorrect beliefs that ART cures HIV/AIDS.

In the developed world, various study designs have been used to investigate the impact of ART availability on risky sexual behaviors. These studies have primarily examined the association between ART-related AB and self-reported sexual behaviors. One longitudinal study observed an association between ART-related AB and incident STIs and HIV in men who have sex with men [15]. Additionally, ecological data have demonstrated riskier sexual behaviors and increased incidence of STIs and HIV among men who have sex with men in San Francisco since the introduction of ART in 1995 [41]. Our study addressed many of the significant limitations to prior studies in the developed world. Our sampling methodology was designed to be representative of the residents of the Kisumu Municipality. As a result, we were able to examine the association of ART-related AB with HIV-seroprevalence in the general population rather than targeting specific high risk groups through methods that did not employ random sampling.

Limitations of this study include the lower than expected response rate, potential bias introduced by 9% of the study population choosing not to provide blood for HIV-testing, and the 17% lower than expected sample size causing a small reduction in power leading to our ability to only detect effects from 1.3 to 1.6 fold. We acknowledge that our sample population represents individuals that could be eventually contacted at home and may not represent everyone who lives in the community. The study team often made greater than the required three visits to contact all eligible household members, and we made visits to the household during the evening and weekend in order to increase enrollment as well as contact individuals not at home during daytime hours (e.g. students and persons employed outside of the home). Despite these efforts, there were many households with no members at home, and due to logistical constraints, we were not able to extend the study to further track household members or sample additional households. Our response rates were lower than the Four African Cities Study [26], perhaps due to differences in recruitment efforts, but we also suspect the growth and urbanization of Kisumu has led to a decreased likelihood of contacting eligible participants at home.

Despite the possible limitations of this study, the results have implications for community education and HIV prevention interventions. The current model of HIV prevention and treatment in Kisumu and much of the world is that HIV prevention programs work within the community, while care and treatment programs provide both HIV treatment and prevention counseling for those who are HIV-positive in medical clinics. Voluntary counseling and testing is the primary referral source that informs HIV-positive persons about the availability of HIV care and treatment programs and the benefits of ART. Our data suggest that there is need for more widespread dissemination of accurate information about ART and for integration of ART-related education with HIV risk-reduction counseling. HIV prevention messages should address changing attitudes about HIV and sexual risk-taking now that ART is becoming widely available.

## Supporting Information

Figure S1 Household and individual response rates
(0.03 MB DOC)

Figure S2 Comparison of HIV seroprevalence among women and men in Kisumu, Kenya 1997 & 2006
(0.03 MB DOC)
