# Supplementary material for: Association of Attitudes and Beliefs towards Antiretroviral Therapy with HIV-Seroprevalence in the General Population of Kisumu, Kenya
Source: PLoS One. 2009 Mar 4;4(3):e4573. doi: 10.1371/journal.pone.0004573 (PMC2649531; doi:10.1371/journal.pone.0004573)
Supplement: Figure S1 — Household and individual response rates (0.03 MB DOC) [file pone.0004573.s001.doc]

**Figure S1:**

Households in original sampling frame

N = 1210

Households participating from original sampling frame

N = 645 (53%)

Replacement households participating*

N = 219

Total number of participating households

N = 864

Total number of persons eligible in these households

N = 3376

Participants enrolled

N = 1655 (91%)

Refused

N = 189 (9%)

Not at home during study visits

N = 1432 (45%)

Gave blood sample

N = 1508 (91%)

Households in sampling frame found at home

N = 708 (59%)

Households refused

N = 63 (9%) of 708

*We attempted to replace each household where we could not find any eligible members at home with the neighboring household. However, we faced similar difficulties finding members at home in replacement households as we did in the originally sampled households. In the end, we enrolled 219 replacement households out of the 502 households that needed replacement.

Total number of persons contacted

N = 1844 (55%)
