# Supplementary material for: Association of Attitudes and Beliefs towards Antiretroviral Therapy with HIV-Seroprevalence in the General Population of Kisumu, Kenya
Source: PLoS One. 2009 Mar 4;4(3):e4573. doi: 10.1371/journal.pone.0004573 (PMC2649531; doi:10.1371/journal.pone.0004573)
Supplement: Figure S2 — Comparison of HIV seroprevalence among women and men in Kisumu, Kenya 1997 & 2006 (0.03 MB DOC) [file pone.0004573.s002.doc]

**Figure S2a**

**Figure S2b**
